# Physical activity and risk of rheumatoid arthritis in women: a population-based prospective study

**DOI:** 10.1186/s13075-015-0560-2

**Published:** 2015-03-04

**Authors:** Daniela Di Giuseppe, Matteo Bottai, Johan Askling, Alicja Wolk

**Affiliations:** Division of Nutritional Epidemiology, Institute of Environmental Medicine, Karolinska Institutet, Nobels vag 13, Stockholm, 171 77 Sweden; Division of Biostatistics, Institute of Environmental Medicine, Karolinska Institutet, Nobels vag 13, Stockholm, 171 77 Sweden; Clinical Epidemiology Unit, Department of Medicine Solna, Karolinska Institutet and Karolinska University Hospital, Stockholm, Sweden

## Abstract

**Introduction:**

Only one study has analysed the association between exercise and development of rheumatoid arthritis (RA), showing no association. Aim of this paper was to evaluate the association of physical activity in all its aspect with RA.

**Methods:**

To examine this association, middle age and elderly women from the Swedish Mammography Cohort, a population-based prospective study, were analysed. Data on physical activity were collected in 1997 by self-administrated food-frequency questionnaire. Risk of RA associated with physical activity was estimated using Cox proportional hazard regression models.

**Results:**

Among 30,112 women born between 1914 and 1948 followed-up from January 1, 2003 to December 31, 2010, 201 RA cases were identified (226,477 person-years). There was a statistically significant 35% lower risk of RA (relative risk (RR), 0.65; 95% confidence interval (CI), 0.43-0.96) among women in the highest category of leisure-time activity (combining more than 20 minute per day of walking/bicycling (median 40–60 minute per day) and more than 1 hour per week of exercise (median 2–3 hours per week)) as compared to women in the lowest category (less than 20 minute per day of walking/bicycling and less than 1 hour per week of exercise). A non-statistically significant decreased risk was observed for household work (−32%) and work/occupation (−15%), while an increased risk was observed for leisure-time physical inactivity (+27%). Daily energy expenditure was not associated with risk of RA.

**Conclusions:**

This prospective population-based cohort study of women supports the hypothesis that physical activity can be a protective factor in the etiology of rheumatoid arthritis. Our results add to accumulated evidence on benefits of modifiable leisure-time physical activity for prevention of many other chronic diseases.

**Electronic supplementary material:**

The online version of this article (doi:10.1186/s13075-015-0560-2) contains supplementary material, which is available to authorized users.

## Introduction

Physical activity is an important health-related behaviour that has been associated with reduced risk of total mortality [1], mainly due to the decrease in risk of cardiovascular diseases [2], type 2 diabetes [3] and even cancer [4]. Evidence is accumulating regarding the beneficial effects of leisure-time physical activity (exercise and walking/bicycling) in reducing inflammation [5-7]. Indeed, previous studies have shown that well-designed physical exercise programmes improve symptoms of rheumatoid arthritis (RA), an autoimmune inflammatory disease [8-11]. However, the only observational study that has evaluated the association between exercise and risk of developing RA has shown no association [12].

The aim of this study is to evaluate the associations between leisure-time physical activity, the part of the daily physical activity that is more easily modifiable, as well as other daily physical activities such as home/household work and work/occupation, leisure-time inactivity (watching television/sitting) and RA development. We have analysed these associations in a prospective population-based cohort study of Swedish women aged 54 to 89 years.

## Methods

### Study population

**Figure 1:**
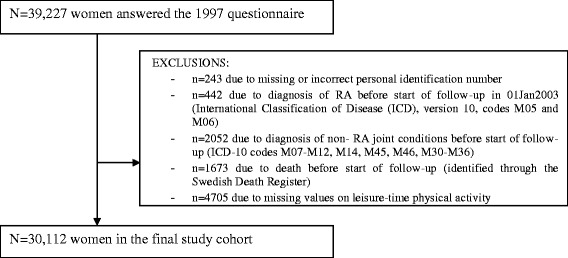
**Flow chart of exclusion from the Swedish Mammography Cohort due to study purpose.** RA, rheumatoid arthritis.

The Swedish Mammography Cohort is a cohort established between 1987 and 1990 when all women residing in Uppsala and Västmanland counties in central Sweden and born between 1914 and 1948 received a questionnaire regarding diet, education and anthropometric factors (weight and height). Of the 66,651 eligible women, 74% responded to the questionnaire. In autumn 1997 a second extended questionnaire was sent to women still alive, with additional questions on physical activity, smoking history and use of dietary supplements. Of the 39,227 questionnaires received (70%), 9,115 were excluded due to reasons described in Figure 1, and the final study cohort included 30,112 women aged 54 to 89. Information on the physical activity exposure and covariates used in the present study was derived from the 1997 questionnaire, since physical activity was not assessed in the 1987 questionnaire. All subjects gave full informed consent to participate in this study [13]. This study was approved by the Regional Research Ethics Board at Karolinska Institutet, and all participants gave their informed consent.

### Assessment of physical activity

Information about physical activity was collected with five questions assessing the usual activity/inactivity level during the previous year with five or six predefined answers (see Additional file 1). The questions focused on work/occupation activity (from mostly sitting down to heavy manual labour), home/household work (from less than 1 hour per day to more than 8 hours per day), leisure-time activities such as walking/bicycling (from hardly ever to more than 1.5 hours per day), exercise (from less than 1 hour per week to more than 5 hours per week) and leisure-time inactivity (watching television/sitting, from less than 1 hour per day to more than 6 hours per day). The question on work/occupation referred to the usual activity performed during the day, either at work or outside work for retired or unemployed women. An additional question regarding sleeping hours was open-ended. A 24-hour energy expenditure score was calculated by adding the products of duration and intensity, expressed as the metabolic equivalent (MET, kcal/kg per hour), for each type of physical activity and inactivity, including sleep [14].

The validity of leisure-time activity and inactivity estimates was assessed comparing the questionnaire with 7-day activity records [14]. The correlations were 0.42 and 0.52 respectively.

### Identification of cases and follow-up of the cohort

Cases of RA were identified through linkage of our study cohort, using the unique Swedish personal identification number, with three different Swedish registers: the Outpatient Register, the Swedish Rheumatology Register and the Inpatient Register. Using these registers, we defined one algorithm to identify incident cases with RA, and a series of alternative definitions in order to investigate the robustness of our main definition. The Outpatient Register of the National Board of Health and Welfare has since 2001 collected information on outpatient visits in nonprimary care (for example, visits to internists or rheumatologists). The Swedish Rheumatology Register is a clinical register that has followed incident RA cases longitudinally as part of standard care since the mid-1990s. The Inpatient Register of the National Board of Health and Welfare started in 1987 and collects virtually complete information on hospitalisation. Chart reviews indicate that approximately 90% of the register-identified cases with RA fulfil the American College of Rheumatology criteria [15,16].

The Inpatient Register was used to identify prevalent cases but not newly diagnosed RA patients during the follow-up period because RA is not a disease that typically leads to hospitalisation in its first stages.

The follow-up period started on 1 January 2003 and ended on 31 December 2010. The delay in the start of the follow-up compared with the start of the Outpatient Register in 2001 was related to the presence of mixed prevalent and incident cases defined as new cases during the first years of this register (see Additional file 2).

### Statistical analysis

We calculated relative risks (RRs) as the hazard rate ratio and their 95% confidence intervals (CIs) to estimate the association between physical activity and RA using the Cox proportional hazard model. All relative risks had age as the time scale [17], and we calculated the age at end of follow-up as age at death, first RA diagnosis or 31 December 2010, whichever was earliest. In the multivariable models we adjusted for age (time scale of the model), cigarette smoking status (categorised as never, former, current ≤10 cigarettes per day or >10 cigarettes per day), alcohol consumption (never, former, current <2 drinks per week or ≥2 drinks per week), body mass index (weight/height^2^ (kg/m^2^), categorised as quartiles) and educational level (<10 years, 10–12 years, >12 years, other). Further adjustment for dairy products and meat consumption, intake of long-chain n-3 polyunsaturated fatty acids, history of diabetes and use of oral contraceptive did not change the RR estimates and therefore these variables were excluded from the final multivariable-adjusted Cox model. The assumption of hazard proportionality [18] was tested for all models and no significant departure from proportionality was observed.

The two variables concerning modifiable leisure-time activity (walking/bicycling and exercise) were analysed both separately and combined.

We performed two sensitivity analyses and evaluated the consistency of our results. First, we delayed the start of follow-up to 1 January 2004 in order to further control for the possible presence of prevalent cases in the register classified in the study as new cases. Among the 29,732 women of the cohort, 159 cases were identified. In the second sensitivity analysis, we included newly diagnosed patients with RA from the Inpatient Register, considering that the exclusion of hospital diagnosed cases in our main analysis might have been too strict. The new cohort consisted of 30,163 women, of which 271 were diagnosed with RA for the first time in the follow-up period 2003 to 2010 (see Additional file 2).

Secondly, we performed a probabilistic sensitivity analysis to evaluate whether the presence of 0 to 20% prevalent cases among the RA cases that we identified as newly diagnosed could have changed our results. This analysis was based on three assumptions of possible different behaviour of prevalent compared with incident cases (see Additional file 2).

All analyses were performed using SAS (version 9.2; SAS Institute, Cary, NC, USA) and Stata (version 12; StataCorp, College Station, TX).

## Results

Among the 30,112 women in the cohort, based on our main definition, 201 were diagnosed with RA for the first time during the follow-up period (mean follow-up time 7.5 years; 226,477 person-years).

We classified leisure-time activity according to minutes of walking/bicycling per day (less or more than 20 minutes per day) and hours of exercise per week (less or more than 1 hour per week). Women in the lowest category of leisure-time activity were older and less educated, with higher body mass index, higher percentage of smokers and lower percentage of drinkers (Table 1).

**Table 1:** **Baseline characteristics of 30,112 women from the Swedish Mammography Cohort, 1997, by leisure-time activity**

	**Leisure-time activity**			
	**Low**	**Intermediate**		**High**
	**Walking**	**Walking**	**Walking**	**Walking**
	**<20 minutes/day**	**<20 minutes/day**	**≥20 minutes/day**	**≥20 minutes/day** ^**a**^
	**Exercise**	**Exercise**	**Exercise**	**Exercise**
	**<1 hour/week**	**≥1 hour/week**	**<1 hour/week**	**≥1 hour/week** ^**a**^
Cases	32	37	14	118
Cohort	3,400	5,369	2,370	18,973
Age (years)	61.6 (9.4)	61.3 (9.0)	59.8 (8.7)	61.3 (8.7)
Years of education (>12 years, %)	15.8	19.9	23.4	19.8
Body mass index (kg/m^2^)	26.2 (4.67)	25.5 (4.11)	24.9 (3.89)	24.7 (3.60)
Current smokers (%)	30.7	23.5	27.6	20.0
Alcohol drinkers (%)	77.9	83.5	84.0	85.0

Data presented as number, mean (standard deviation) or percentage. ^a^Median of walking/bicycling in the category ≥20 was 40 to 60 minutes per day; median of exercise in the category ≥1 was 2 to 3 hours per week.

Leisure-time activity was calculated by combining information about walking/bicycling and exercise (Table 2). The age-adjusted relative risk of developing RA comparing women in the higher category of leisure-time activity (more than 20 minutes per day of walking/bicycling and more than 1 hour per week of exercise) with women in the lower category (less than 20 minutes per day of walking/bicycling and less than 1 hour per week of exercise) was 0.62 (95% CI, 0.42 to 0.92), while the multivariable-adjusted relative risk was 0.65 (95% CI, 0.43 to 0.96). We had insufficient statistical power to evaluate the association with higher leisure-time physical activity levels since only 7% of women in the cohort walked more than 1.5 hours per day (16 cases) and only 11.3% exercised more than 5 hours per week (22 cases). The rate of RA was lower among women in the highest category of leisure-time activity (7.96 per 10,000 person-years) than among women in the lower category (12.91 per 10,000 person-years). We calculated the population prevented fraction, the equivalent of the population attributable risk when analysing a protective factor, and 0.22 was the proportion of the hypothetical total load of disease that had been prevented by exposure to the higher category of leisure-time activity.

**Table 2:** **Age-adjusted and multivariable-adjusted relative risk** ^a^ **of rheumatoid arthritis by leisure-time activity in the Swedish Mammography Cohort, follow-up 2003 to 2010**

**Exercise**	**Walking/bicycling**			
	**Cases/cohort**	**<20 minutes/day**	**Cases/cohort**	**≥20 minutes/day**
				**(median 40 to 60)**
**Age-adjusted**				
<1 hour/week	32/3,400	1.00 (reference)	14/2,370	0.57
				(0.31 to 1.07)
≥1 hour/week	37/5,369	0.70	118/18,973	0.62^b^
(median 2 to 3)		(0.44 to 1.12)		(0.42 to 0.92)
**Multivariable-adjusted** ^**a**^				
<1 hour/week	32/3,400	1.00 (Ref)	14/2,370	0.59
				(0.31 to 1.10)
≥1 hour/week	37/5,369	0.72	118/18,973	0.65^b^
(median 2 to 3)		(0.45 to 1.16)		(0.43 to 0.96)

Data in parentheses are the 95% confidence interval. ^a^Adjusted for age (continuous), smoking status (categorised as never, former, current ≤10 cigarettes per day or >10 cigarettes per day), alcohol intake (never, former, current <2 drinks per week, ≥2 drinks per week), body mass index (quartiles) and educational level (<10 years, 10–12 years, >12 years, other). ^b^Corresponding to a median of 40 to 60 minutes per day of walking/bicycling and 2 to 3 hours per week of exercise.

Although none of the single physical activities analysed separately were statistically significantly associated with the risk of RA (Table 3), we observed a decrease in risk with hours of home/household work (32% decrease for more than 6 hours per day), hours of exercise per week (20% decrease for 2 hours or more per week), walking/standing at work (15% decrease compared with sitting) and duration of walking/bicycling per day (9% for 20 minutes or more per day). On the other hand, we observed an increased risk among women in the high category of inactive leisure-time (27% increase for more than 2 hours of watching television/sitting per day). Daily energy expenditure was not significantly associated with risk of RA when categorised into quartiles. To evaluate the RA risk among women with very high and very low energy expenditure we performed a second analysis using the 5th percentile (35.3 MET-hours per day) and the 95th percentile (50.4 MET-hours per day) as cutoff values. We observed that women with a daily energy expenditure of less than 35.3 MET-hours per day had a 75% increased risk of RA (RR, 1.75; 95% CI, 0.97 to 3.17).

**Table 3:** **Relative risk of rheumatoid arthritis by different activity status among women from the Swedish Mammography Cohort, follow-up 2003 to 2010**

	**Person-years**	**Number of cases** ^**a**^ **/women in the cohort**	**Age-adjusted relative risk**	**95% CI**	**Multivariable** ^**b**^ **relative risk**	**95% CI**
**Leisure-time activity**						
*Walking/bicycling*						
<20 minutes/day	64,615	69/8,769	1.00		1.00	
≥20 minutes/day (median 40 to 60)	161,862	132/21,343	0.91	0.76 to 1.08	0.91	0.77 to 1.09
*Exercise*						
<1 hour/week	42,398	46/5,770	1.00		1.00	
1 hour/week	54,190	48/7,150	0.80	0.54 to 1.20	0.83	0.55 to 1.25
≥2 hours/week (median 2 to 3)	129,889	107/17,192	0.77	0.55 to 1.10	0.80	0.56 to 1.14
**Leisure-time inactivity**						
*Watching television/sitting*						
<1 hour/day	25,361	21/3,280	1.00		1.00	
1 to 2 hours/day	104,213	93/13,665	1.15	0.71 to 1.84	1.11	0.69 to 1.78
>2 hours/day (median 3 to 4)	95,068	87/12,922	1.37	0.84 to 2.24	1.27	0.77 to 2.08
**Other daily physical activities**						
*Home/household work*						
<2 hours/day	86,311	83/11,364	1.00		1.00	
3 to 4 hours/day	77,696	74/10,344	1.05	0.76 to 1.43	1.04	0.75 to 1.43
5 to 6 hours/day	33,313	24/4,458	0.84	0.53 to 1.33	0.84	0.53 to 1.35
>6 hours/day (median 7 to 8)	22,131	13/2,981	0.67	0.37 to 1.22	0.68	0.37 to 1.24
*Work/occupation*						
Sitting	107,145	103/14,290	1.00		1.00	
Walking/standing	105,432	87/13,917	0.86	0.64 to 1.14	0.85	0.63 to 1.13
**Total energy expenditure (24 hours)**						
<38.8 MET-hours per day	48,847	45/6,567	1.00		1.00	
38.8 to 42.2 MET-hours per day	51,492	47/6,792	1.00	0.66 to 1.50	1.01	0.67 to 1.53
42.2 to 45.9 MET-hours per day	51,285	44/6,800	0.94	0.62 to 1.43	0.96	0.63 to 1.46
≥45.9 MET-hours per day	52,099	46/6,851	0.97	0.64 to 1.46	0.97	0.64 to 1.47

CI, confidence interval; MET, metabolic equivalent (kcal/kg per hour). ^a^Number of cases do not add to the total (*n* = 201) due to missing values for information about the single physical activities. ^b^Adjusted for age (continuous), smoking status (categorised as never, former, current ≤10 cigarettes per day or >10 cigarettes per day), alcohol intake (never, former, current <2 drinks per week, ≥2 drinks per week), body mass index (quartiles) and educational level (<10 years, 10–12 years, >12 years, other).

We further examined the association of the modifiable leisure-time activity (combined walking/bicycling and exercise) and RA in two sensitivity analyses. When we delayed the start of follow-up to 1 January 2004, we observed that the risk of RA was 42% lower among women in the highest category of leisure-time activity (RR, 0.58; 95% CI, 0.38 to 0.90). In the second sensitivity analysis, we included newly diagnosed RA patients through the Inpatient Register during the period 1 January 2003 until 31 December 2010. The results were consistent with the results from the main analysis (RR for women in the highest category, 0.71; 95% CI, 0.50 to 1.01). The estimates from the probabilistic sensitivity analysis did not differ from the main analysis (see Additional file 2), indicating that the results were not affected by the possible presence of prevalent cases analysed as newly diagnosed cases.

## Discussion

In this prospective population-based cohort study we observed an inverse association between different daily physical activities and risk of RA. In particular, modifiable leisure-time activity (daily walking/bicycling >20 minutes and weekly exercise >1 hour) was statistically significantly inversely associated (35% decrease in risk) with RA. Daily energy expenditure was not associated with risk of RA.

### Comparison with other studies

Only one previous study based on self-report and subsequently validated cases (*n* = 158) examined the association between physical activity and risk of developing RA [12]. The prospective cohort study, which reported a mild, inverse nonstatistically significant association, assessed physical activity using only a three-level exercise score (low, medium, high) and had limited power.

Our results add to the accumulating evidence on benefits of leisure-time physical activity for prevention of many chronic diseases such as coronary heart disease, high blood pressure, type 2 diabetes, metabolic syndrome, colon cancer, breast cancer, prostate cancer and depression, and all-cause mortality [19,20]. Guidelines from the World Health Organization recommend that adults should perform at least 150 minutes per week of moderate-intensity aerobic physical activity [19]. Our findings support this recommendation. Moreover, our results indicate that not only isolated training sessions, but a lifestyle with sufficient physical activity on different levels is important in the prevention of RA.

### Biological mechanism

We can only speculate about the mechanisms behind the beneficial effect of physical activity, but it has been linked to a decrease in chronic inflammation [21], and an increase in innate immune function [22]. The anti-inflammatory effect of physical activity may further be related to loss of body fat, reductions in macrophage accumulation in adipose tissue, muscle production of interleukin-6 and irisin, secretion of adiponectin from adipose tissue into the bloodstream, or alterations in the balance between the sympathetic and parasympathetic nervous system [5,7,23].

In particular, it has been observed that exercise reduces C-reactive protein [24,25] and tumour necrosis factor alpha [26,27] levels, two of the biomarkers for RA. It has been hypothesised that exercise training reduces C-reactive protein levels by reducing cytokine production in fat and by increasing insulin sensitivity [28]. Moreover, exercise increases levels of epinephrine and interleukin-6, which appears to inhibit endotoxin-induced appearance of tumour necrosis factor alpha with an independent mechanism [29]. Whereas these immunological changes may lead to an amelioration of existing RA symptoms, our study raises the hypothesis that they may also protect from clinical onset. In support of this hypothesis, it is important to underline that physical activity is a protective factor in many inflammation-related diseases, such as cardiovascular diseases [30], type 2 diabetes [31] and cancer [32-34].

### Strengths and limitation

The main strength of this study was the prospective population-based design and the almost complete follow-up of the participants through Swedish registers. We were able to measure all major types of daily physical activity with relatively high validity [14]. The assessment of the exposure in 1997, more than 5 years before the start of follow-up, prevented possible differential misclassification of exposure due to change in physical activities related to RA presymptoms, which could lead to reverse causation.

We delayed the start of follow-up from 1997 (date of collection of the exposure information) to 2003 since the Outpatient Register only started in 2001 and during the first years it collected both incident and prevalent cases as newly diagnosed RA patients (further details published earlier [35]). We performed a sensitivity analysis with one additional year of delay in the follow-up start (1 January 2004), and the results were consistent. In our analyses, we excluded cases of RA identified through the Inpatient Register even after the start of the follow-up, because RA is usually a disease that does not lead to hospitalisation during first stages. In a second sensitivity analysis we included those hospitalised RA cases identified in the Inpatient Register, and the results were similar but no longer significant.

Among the limitations of the present study, because of the observational design, we cannot exclude the possibility of residual and unmeasured confounding. The association of physical activity with RA risk could be underestimated due to misclassification of the exposure, considering that different individuals could perform the same activity with different intensity. Despite the relatively high validity of physical activity measures, there is also the possibility of nondifferential misclassification of the exposure since women who consider physical activity a healthy behaviour might over-report it. We also need to consider that lack of physical activity can be due to health problems, and that these problems, instead of the physical inactivity, are associated with increased risk of RA. Despite measures to evaluate misclassification of prevalent RA as incident RA cases (see Additional file 2), we cannot exclude misclassification of RA with other diseases with a longer prediagnostic period than that typically seen in RA, such as osteoarthritis. We did not have information on the presence of anti-citrullinated protein antibody or rheumatoid factor to perform stratified analyses on subtypes of RA. Moreover, the results cannot be generalised to younger women and men.

## Conclusion

In this prospective cohort study we observed a lowered RA risk among women who were physically active during leisure time (>20 minutes per day of walking/bicycling and >1 hour per week of exercise). Other kinds of physical activities, including home/household work and occupation during the day, were also observed to decrease the risk of RA, while leisure-time inactivity increased the risk. The observed level of the modifiable leisure-time physical activity to achieve beneficial effects for RA is in accordance with findings regarding the prevention of cardiovascular diseases, type 2 diabetes, cancer and mortality, and supports the World Health Organization recommendation.

## Additional files

Additional file 1:
**Is a figure presenting the section of the questionnaire containing the questions regarding physical activity.**
Additional file 2:
**Is an appendix presenting results from sensitivity analyses.**

## Abbreviations

CI: confidence interval
MET: metabolic equivalent
RA: rheumatoid arthritis
RR: relative risk
