# Supplementary material for: Physical activity and risk of rheumatoid arthritis in women: a population-based prospective study
Source: Arthritis Res Ther. 2015 Mar 4;17(1):40. doi: 10.1186/s13075-015-0560-2 (PMC4365521; doi:10.1186/s13075-015-0560-2)
Supplement: Additional file 1: — Is a figure presenting the section of the questionnaire containing the questions regarding physical activity. [file 13075_2015_560_MOESM1_ESM.pdf]

PHYSICAL ACTIVITY AND EXERCISE

10. Mark your level of physical activity **at different ages**:

**Home/householdwork**	15 yrs	30 yrs	50 yrs	this yr
Less than 1 hour/day	☐	☐	☐	☐
1-2 hours/day	☐	☐	☐	☐
3-4 hours/day	☐	☐	☐	☐
5-6 hours/day	☐	☐	☐	☐
7-8 hours/day	☐	☐	☐	☐
More than 8 hours/day	☐	☐	☐	☐

**Walking/cycling**	15 yrs	30 yrs	50 yrs	this yr
Hardly ever	☐	☐	☐	☐
Less than 20 min/day	☐	☐	☐	☐
20-40 minutes/day	☐	☐	☐	☐
40-60 minutes/day	☐	☐	☐	☐
1-1,5 hours/day	☐	☐	☐	☐
More than 1,5 hours/day	☐	☐	☐	☐

☐

**Work/occupation**	15 yrs	30 yrs	50 yrs	this yr
Mostly sitting down	☐	☐	☐	☐
Sitting down half the time	☐	☐	☐	☐
Mostly standing up	☐	☐	☐	☐
Mostly walking, lifts, carry little	☐	☐	☐	☐
Mostly walking, lifts,carry much	☐	☐	☐	☐
Heavy manual labour	☐	☐	☐	☐

Leisure time

**Watching TV/reading**	15 yrs	30 yrs	50 yrs	this yr
Less than 1 hour/day	☐	☐	☐	☐
1-2 hours/day	☐	☐	☐	☐
3-4 hours/day	☐	☐	☐	☐
5-6 hours/day	☐	☐	☐	☐
More than 6 hours/day	☐	☐	☐	☐
Exercise				
Less than 1 hour/week	☐	☐	☐	☐
1 hour/week	☐	☐	☐	☐
2-3 hours/week	☐	☐	☐	☐
4-5 hours/week	☐	☐	☐	☐
More than 5 hours/week	☐	☐	☐	☐

11. How many hours in 24 hours do you usually...

Sleep ☐☐ hours/ 24 hours   ☐☐ hours/ 24 hours
