# Supplementary material for: Physical activity and risk of rheumatoid arthritis in women: a population-based prospective study
Source: Arthritis Res Ther. 2015 Mar 4;17(1):40. doi: 10.1186/s13075-015-0560-2 (PMC4365521; doi:10.1186/s13075-015-0560-2)
Supplement: Additional file 2: — Is an appendix presenting results from sensitivity analyses. [file 13075_2015_560_MOESM2_ESM.pdf]

Appendix

A) Alternative outcome definitions

Start of follow-up

The period of follow-up for this study started January 1, 2003 and ended December 31, 2010. The delay on the start of the follow-up compared to the date of the questionnaire (1997) was due to the start of the Outpatient Register only in 2001. Moreover, we considered a 2-year period of wash-out to avoid the inclusion of prevalent RA cases as newly diagnosed patients. This decision was based on the observation of the distribution of new cases per year in the Outpatient Register (Fig App-1, see below).

Figure App-1: Frequency of newly diagnosed RA cases identified through the Outpatient Register between 2001 and 2010.

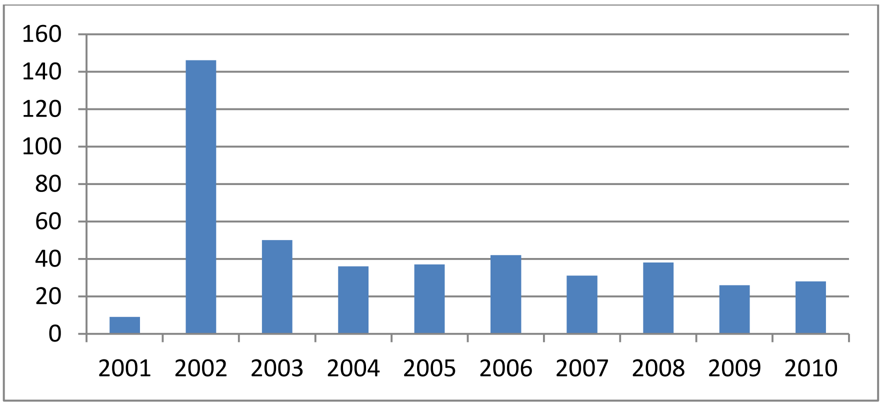

However, we also considered a longer wash-out period in sensitivity analysis.

Figure App-2: Timeline to illustrate starts of follow-up according to the main analysis and to the different sensitivity analyses performed.

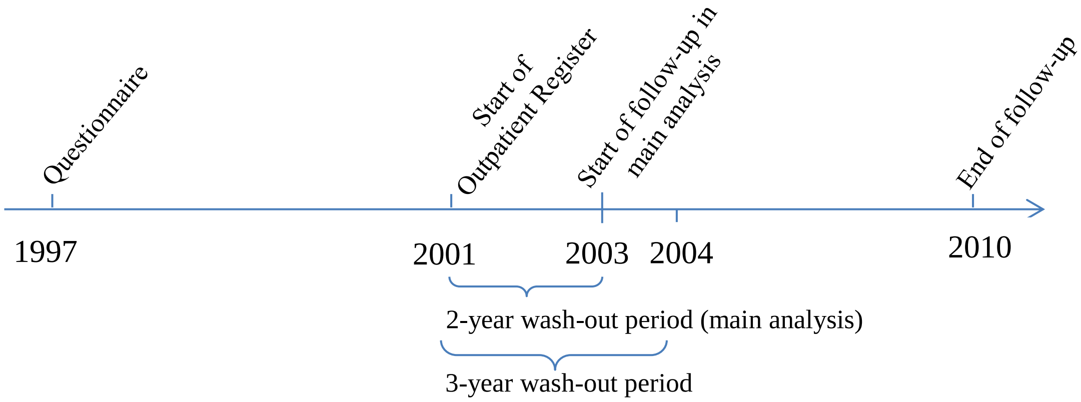

Results on leisure-time activity (walking/bicycling and exercise) are reported in Table A for a 3-year wash-out period and were consistent with main analysis.

<u>Use of Inpatient Register</u>

In the study data on hospitalizations from the Inpatient Register was used only to identify prevalent RA cases at start of follow-up. In a sensitivity analyses (Table A, b) we also included newly diagnosed RA patients identified through the Inpatient Register. Results for the follow-up periods 2003-2010 were concordant with main analysis.

TABLE A. Multivariable adjusted relative risk‡ and 95 percent confidence interval (in parenthesis) for four additional alternative case definitions (different time of follow-up and exclusion/inclusion of the Inpatient Register) by leisure-time activity levels in the Swedish Mammography Cohort.

				Sensitivity a		Sensitivity b
	Cases	Main Analysis 2-years w-o*	Cases	Follow-up 2004-2010 3-year w-o*	Cases	Follow-up 2003-2010 + Inpatient Reg.
**Number of cases**	**201**		**159**		**271**	
Leisure-time activity						
**Walking <20 min/day Exercise <1 hour/week**	32	1.00(ref)	28	1.00(ref)	41	1.00(ref)
**Walking ≥20 min/day Exercise <1 hour/week**	14	0.59 (0.31-1.10)	11	0.51 (0.25-1.04)	18	0.61 (0.35-1.07)
**Walking <20 min/day Exercise ≥1 hour/week**	37	0.72 (0.45-1.16)	25	0.55 (0.32-0.95)	46	0.70 (0.46-1.07)
**Walking ≥20 min/day Exercise ≥1 hour/week**	118	0.65 (0.43-0.96)	95	0.58 (0.38-0.90)	166	0.71 (0.50-1.01)

‡ Adjusted for age (continuous), smoking status (categorized as never, former, current ≤10 cigarettes/day or >10 cigarettes/day), alcohol intake (never, former, current <2 drinks per week, ≥2 drinks per week), body mass index (quartiles), and educational level (<10, 10-12, >12, other).
*w-o = wash-out period

B) Exclusion of potential prevalent cases

Despite our attention on avoiding the inclusion in the analysis of prevalent cases, is still possible that some newly diagnosed RA patients identified during the follow-up period have developed the disease before the start of follow-up. To evaluate the possible changes in the estimates due to the presence of prevalent cases, we performed a probabilistic sensitivity analysis. We assumed an a priori distribution that captures our uncertainty about the amount of prevalent cases in the cohort, in the form of a uniform with values 0 to 20. We then performed simulations with 200 draws from the uniform distribution: each draw corresponded to the percentage of prevalent cases that should be randomly excluded. We then calculated the corresponding RR for leisure-time activity. We obtained the distributions of the RR estimates according to three assumptions (median and standard deviation are reported in table B):

1- Prevalent cases did not change their physical activity habits (cases were excluded randomly from all categories)
2- All prevalent cases decreased their physical activity level (cases were excluded only from the lower category)
3- All prevalent cases increased their physical activity level (cases were excluded only from the upper category)

Results from the sensitivity analyses did not differ from the main analysis.

TABLE B. Median values‡ and standard deviation of the distributions of RA relative risks by leisure-time activity levels according to three alternative assumptions about the behavior of prevalent cases among incident cases (1. Prevalent cases did not change their physical activity habits; 2. Prevalent cases decreased their physical activity level; 3. Prevalent cases increased their physical activity level).

Leisure-time activity	**Assumption 1**	**Assumption 2**	**Assumption 3**
**Walking <20 min/day Exercise <1 hour/week**	1.00	1.00	1.00
**Walking ≥20 min/day Exercise <1 hour/week**	0.58 ± 0.06	0.63 ± 0.03	0.59 ± 0.003
**Walking <20 min/day Exercise ≥1 hour/week**	0.72 ± 0.06	0.77 ± 0.04	0.72 ± 0.003
**Walking ≥20 min/day Exercise ≥1 hour/week**	0.64 ± 0.04	0.69 ± 0.04	0.60 ± 0.014

‡ Adjusted for age (continuous), smoking status (categorized as never, former, current ≤10 cigarettes/day or >10 cigarettes/day), alcohol intake (never, former, current <2 drinks per week, ≥2 drinks per week), body mass index (quartiles), and educational level (<10, 10-12, >12, other).
